# Highly conserved composite transposon harbouring aerobactin *iuc3* in *Klebsiella pneumoniae* from pigs

**DOI:** 10.1099/mgen.0.000960

**Published:** 2023-02-23

**Authors:** Håkon Kaspersen, Fiona Valerie Franklin-Alming, Marit A. K. Hetland, Eva Bernhoff, Iren H. Löhr, Jatesada Jiwakanon, Anne Margrete Urdahl, Thongpan Leangapichart, Marianne Sunde

**Affiliations:** ^1^​ Norwegian Veterinary Institute, Ås, Norway; ^2^​ Department of Medical Microbiology, Stavanger University Hospital, Stavanger, Norway; ^3^​ Department of Biological Sciences, Faculty of Mathematics and Natural Sciences, University of Bergen, Bergen, Norway; ^4^​ Department of Clinical Science, Faculty of Medicine, University of Bergen, Bergen, Norway; ^5^​ Khon Kaen University, Khon Kaen, Thailand

**Keywords:** aerobactin, composite transposon, *Klebsiella pneumoniae*, Norway, pigs, Thailand

## Abstract

*
Klebsiella pneumoniae* is an important opportunistic pathogen associated with severe invasive disease in humans. Hypervirulent *K. pneumoniae *, which are *K. pneumoniae* with several acquired virulence determinants such as the siderophore aerobactin and others, are more prominent in countries in South and South-East Asia compared to European countries. This *Klebsiella* pathotype is capable of causing liver abscesses in immunocompetent persons in the community. *K. pneumoniae* has not been extensively studied in non-human niches. In the present study, *K. pneumoniae* isolated from caecal samples (*n*=299) from healthy fattening pigs in Norway were characterized with regard to population structure and virulence determinants. These data were compared to data from a previous study on *K. pneumoniae* from healthy pigs in Thailand. Lastly, an in-depth plasmid study on *K. pneumoniae* with aerobactin was performed. Culturing and whole-genome sequencing was applied to detect, confirm and characterize *K. pneumoniae* isolates. Phylogenetic analysis described the evolutionary relationship and diversity of the isolates, while virulence determinants and sequence types were detected with Kleborate. Long-read sequencing was applied to obtain the complete sequence of virulence plasmids harbouring aerobactin. A total of 48.8 % of the investigated Norwegian pig caecal samples (*n*=299) were positive for *K. pneumoniae *. Acquired virulence determinants were detected in 72.6 % of the isolates, the most prominent being aerobactin (69.2 %), all of which were *iuc3*. In contrast, only 4.6 % of the isolates from Thailand harboured aerobactin. The aerobactin operon was located on potentially conjugative IncFIB_K_/FII_K_ plasmids of varying sizes in isolates from both countries. A putative, highly conserved composite transposon with a mean length of 16.2 kb flanked by truncated IS*3*-family IS*407*-group insertion sequences was detected on these plasmids, harbouring the aerobactin operon as well as several genes that may confer increased fitness in mammalian hosts. This putative composite transposon was also detected in plasmids harboured by *K. pneumoniae* from several countries and sources, such as human clinical samples. The high occurrence of *K. pneumoniae* harbouring aerobactin in Norwegian pigs, taken together with international data, suggest that pigs are a reservoir for *K. pneumoniae* with *iuc3*. Truncation of the flanking ISKpn78-element suggest that the putative composite transposon has been permanently integrated into the plasmid, and that it is no longer mobilizable.

## Data Summary

The raw Illumina reads, Nanopore fast5 files and the 16 complete genomes have been uploaded to the National Center for Biotechnology Information (NCBI) under the BioProject accession number PRJNA835677. Tables S1 and S2 (available with the online version of this article) show detailed information.

**Impact Statement**

*
Klebsiella pneumoniae* belongs to the ESKAPE pathogens (* Enterococcus faecium *, *Staphylococcus aureus *, *K. pneumoniae *, *Acinetobacter baumannii *, *Pseudomonas aeruginosa *, *Enterobacter* spp.), which are important causes of hospital-acquired infections. *K. pneumoniae* is a well-studied human pathogen and antimicrobial-resistant, high-risk clones and hypervirulent clones have emerged globally. *K. pneumoniae* is also present in a variety of environmental niches, but currently there is a lack of knowledge on the occurrence and characteristics of *K. pneumoniae* from non-human sources. Certain environments are associated with a high *K. pneumoniae* load, and *K. pneumoniae* in these niches may constitute a reservoir for transmission of strains and genetic elements. Here, we characterize *K. pneumoniae* from the healthy fattening pig population in Norway. Furthermore, we compared *K. pneumoniae* from Norwegian pigs to another dataset from pigs in Thailand obtained from one of our previous studies. We detected a high occurrence (~80 %) of the virulence operon aerobactin lineage *iuc3* among the isolates from Norway. We also observed *iuc3* among *K. pneumoniae* from Thailand, but at lower occurrence. This virulence operon is regarded as one of the most important virulence determinants in *K. pneumoniae* and is highly associated with disease in humans. We performed in-depth genomic investigations, based on data from extensive short- and long-read sequencing, and identified plasmids belonging to the IncF incompatibility group as vectors for the *iuc3* aerobactin operon. Furthermore, we identified the aerobactin operon on a highly conserved putative composite transposon with additional genes that may give the host bacterium fitness advantages in the mammalian gut environment. Truncated insertion sequence elements flanked the putative composite transposon, suggesting permanent integration into the plasmid. Comparative genomics revealed the presence of this conserved composite transposon in plasmids disseminated worldwide, obtained from both *K. pneumoniae* causing bloodstream infections in humans and from clinical isolates from animals. Our results suggest that the composite transposon harbouring aerobactin is permanently integrated into potentially mobile and diverse IncF plasmids. The association of pigs and the aerobactin lineage *iuc3* suggests that pigs may be a reservoir for *K. pneumoniae* harbouring *iuc3*. Our findings provide new insight into a key virulence determinant in *K. pneumoniae* and extend current knowledge about this important bacterial pathogen.

## Introduction

*Klebsiella pneumoniae* is an important opportunistic pathogen listed on the World Health Organization (WHO) priority list, and urgently requires development of new control strategies [1]. *K. pneumoniae* consists of a group of closely related subspecies, collectively called the *K. pneumoniae* species complex (KpSC) [2]. The most prominent subspecies in the KpSC is *K. pneumoniae sensu stricto*, which also seems to have the highest clinical importance [3]. Infections with *K. pneumoniae sensu stricto*, hereafter referred to as *K. pneumoniae *, are generally divided into hospital-acquired and community-acquired infections. The hospital-acquired infections are often associated with multi-drug resistant *K. pneumoniae *, while the community-acquired infections are in some settings more often associated with hypervirulent strains that can cause severe infections such as sepsis and liver abscesses, among others. Convergence events, where hypervirulent strains acquire resistance determinants, have increasingly been reported worldwide, and this is a cause for concern due to the limited treatment options [4–6].

Hypervirulent strains typically harbour several virulence determinants, such as aerobactin, salmochelin, yersiniabactin and/or the gene *rmpA*/*rmpA2*. Aerobactin, salmochelin and yersiniabactin are encoded on gene clusters that synthesize siderophore systems that scavenge iron from the host. The presence of *rmpA*/*rmpA2* genes is associated with capsule overproduction [7]. Aerobactin has previously been identified as a major virulence determinant in *K. pneumoniae *, and is often associated with hypervirulence [8, 9]. Aerobactin alone seems to contribute to over 90 % of the total siderophore production, even if other siderophores are present [10]. Several lineages of aerobactin exist, some of which are associated with conserved plasmids, such as the aerobactin lineages *iuc1* and *iuc2* located on the KpVP-1 and KpVP-2 virulence plasmids [11]. The remaining lineages are highly associated with diverse plasmid structures, except *iuc4*, which appears to be restricted to the *K. pneumoniae* subsp. *rhinoscleromatis* chromosome [11]. Aerobactin *iuc3* is reportedly mobilized on diverse, potentially conjugative IncF plasmids among members of the KpSC [11–13].

*K. pneumoniae* is a well-studied human pathogen. However, there are considerable knowledge gaps regarding *K. pneumoniae* from animal reservoirs. *K. pneumoniae* have been found to be the causative agent for seasonal outbreaks of septicaemia in pigs in England [14], and an association between *K. pneumoniae* and the aerobactin lineage *iuc3* has been described from pigs in Germany and Italy [12, 15]. Moreover, a study by Leangapichart *et al.* investigated transmission of *K. pneumoniae* between pigs and humans on farms in Thailand and identified potential zoonotic transmission [16]. Aerobactin lineage *iuc3* was detected in some of the Thai pig isolates. Altogether, these studies indicate that pigs may be a reservoir for *K. pneumoniae* harbouring aerobactin, with a risk of possible zoonotic transmission.

In the present study, KpSC isolates from healthy Norwegian pigs were characterized with regards to their virulence genes, genomic diversity and population structure, and compared to KpSC isolates from pigs in Thailand (from the study by Leangapichart *et al.*) [16]. Lastly, an in-depth study of genomic elements harbouring aerobactin was performed, including comparisons to genomic data from other countries.

## Methods

### Sampling and isolate detection

In 2019, caecal samples from fattening pigs sampled at slaughter were included in the NORM-VET surveillance programme [17]. Only one pig per herd was included. These samples were available for the current study, and were screened for the presence of *Klebsiella* spp*.* Each sample was plated directly onto Simmons citrate agar with 1 % inositol (SCAI; Oxoid) and incubated at 37 °C for 48 h. Presumptive *Klebsiella* spp*.* colonies were selected based on morphology, and confirmed as *Klebsiella* spp*.* with a MALDI-TOF instrument (Bruker Daltonik).

### DNA extraction and Illumina sequencing

DNA extraction from pure cultures of the Norwegian KpSC pig isolates was performed by using a MagNA Pure 96 instrument (Roche) with the DNA/Viral NA SV 2.0 kit and the pathogen universal 200 4.0 protocol. Sequencing library preparation was performed by using the Illumina Nextera DNA Flex library prep kit (Illumina), followed by sequencing on an Illumina MiSeq instrument, producing paired-end reads with a length of 300 bp.

The genomes of the KpSC originating from pig isolates from Thailand were available from a previous study [16]. A total of 87 genomes were included.

### Quality control and draft assemblies

All raw reads were quality controlled by using FastQC (https://www.bioinformatics.babraham.ac.uk/projects/fastqc/). Trim Galore (https://www.bioinformatics.babraham.ac.uk/projects/trim_galore/) version 0.6.4 was used to trim adapter sequences and low-quality nucleotides. Unicycler [18] version 0.4.8 was subsequently used to assemble the genomes using the trimmed reads and default settings. Quast [19] version 5.0.2 was used to determine the quality of the assemblies.

### Multilocus sequence typing and virulence and resistance gene detection

Kleborate [20] version 2.1.0 was used to identify the exact species and sequence types (STs) of the isolates, as well as the presence of virulence and resistance genes. Kleborate also reports the STs of each virulence operon, e.g. aerobactin ST (AbST) and yersiniabactin ST (YbST). The species assignment of the isolates from Thailand was performed previously [16], while the virulence and resistance gene detection and multilocus sequence typing were carried out in the current study with Kleborate version 2.1.0.

### Pangenome and phylogenetic analysis

The alppaca pipeline [21] version 0.4.1 (DOI: 10.5281/zenodo.4452122) was used to generate a core-gene phylogeny. Briefly, all draft assemblies were annotated using Prokka [22] version 1.14.5. Panaroo [23] version 1.2.2 was used to determine the pan genome of the isolates using the sensitive mode, and to generate a core-gene alignment with mafft [24] version 7.464. iq-tree [25] version 1.6.12 was used to generate a maximum-likelihood phylogeny from the core-gene alignment, using ModelFinder plus [26] and 1000 ultrafast bootstrap replicates [27]. Finally, snp-dists (https://github.com/tseemann/snp-dists) version 0.6.3 was used to determine the pairwise SNP distances from the core-gene alignment. The phylogenetic tree was visualized in R [28] version 4.0.5 using the packages ggtree [29] version 3.0.4 and ggtreeExtra [30] version 1.0.4.

### Long-read sequencing and assembly

Isolates that carried aerobactin were considered for long-read sequencing. To capture the diversity of the putative aerobactin-encoding plasmids, 16 isolates were selected for Oxford Nanopore sequencing based on the diversity in STs and AbSTs, 4 of which were from Thailand. DNA was extracted from pure cultures using the GenFind v3 kit (Beckman Coulter Life Sciences) on a Biomek i7 instrument, using the protocol ‘DNA extraction from bacteria using GenFind v3’. Library preparation was done using the SQK-LSK109 ligation sequencing kit. The sequencing was performed on an Oxford Nanopore GridION instrument (Oxford Nanopore Technologies), using a MinION R9.4.1 flow cell. Guppy version 5.0.14 (Oxford Nanopore Technologies) was used for basecalling and demultiplexing, using the super-accuracy basecalling model.

The genomes that were both Illumina and Nanopore sequenced were subjected to hybrid assembly. First, the long reads were quality controlled with NanoPlot [31] version 1.33.1. Then, Filtlong [32] version 0.2.0 was used to discard the lowest 10 % of reads based on length and quality. Unicycler was used to generate hybrid assemblies based on the filtered long reads and untrimmed Illumina reads. If the hybrid assembly failed, Filtlong was run again and set to remove the lowest 20 % of reads.

Genomes that were either incomplete after hybrid assembly or failed to assemble twice were subjected to long-read assembly and consensus analysis using Trycycler [33] version 0.5.1. Briefly, Trycycler was used to generate 12 subsets of reads, where each set of four subsets were independently assembled using Minipolish [34] version 0.1.2, Flye [35] version 2.9 and Raven (https://github.com/lbcb-sci/raven) version 1.6.1. Then, the contigs were clustered, and potential outliers were removed. If a cluster was represented by less than four contigs, a new subset of 24 read sets were generated and assembled as above. The contig clusters were then reconciled and aligned, before a consensus was made. The resulting long-read assembly was polished by using Medaka (Oxford Nanopore Technologies) version 1.4.4 and two rounds of Pilon [36] version 1.23.

### Plasmid detection, characterization and comparison

All complete assemblies were run through the Ellipsis pipeline (DOI: 10.5281/zenodo.4563897) to characterize incompatibility types, virulence genes and resistance genes on closed plasmid sequences. Briefly, MOB-suite [37] version 3.0.1 was run to classify each contig as plasmid or chromosome, and to detect incompatibility types. Each detected plasmid sequence was subjected to ResFinder [38] (database downloaded on February 11th 2020), VirulenceFinder [39] (database downloaded on March 6th 2020) and PlasmidFinder [40] (database downloaded on March 6th 2020) for acquired resistance gene detection, virulence gene detection and replicon typing, respectively. To facilitate the detection of aerobactin with VirulenceFinder, all aerobactin allele sequences from Kleborate were added to the VirulenceFinder database. Plasmids that harboured aerobactin were annotated by using Bakta [41] version 1.3.3 with the Bakta database version 3.1, using the --complete option, in addition to manual curation.

The aerobactin-harbouring plasmids (*n*=16) were compared to three closely related plasmids, detected with mash by MOB-suite. These plasmids were previously isolated from humans with bloodstream infections in Laos (accession no. MK649829) and Vietnam (accession no. MK649826), and from a pig in Thailand (accession no. CP041094). All three sequences were annotated using Bakta as described above.

Aerobactin-harbouring plasmids were subjected to IncF replicon sequence typing (RST) [40] version 0.1.0 on the Center of Genomic Epidemiology website (https://cge.cbs.dtu.dk/services/pMLST/).

Minimap2 [42] version 2.22 was used to compare the sequence similarity between all 19 aerobactin-harbouring plasmids, using the all-against-all mode with 0.1 as the minimum secondary-to-primary score ratio. The plasmid fasta files were indexed with Seqkit version 0.12.0, using the faidx command and default settings. The indexes, annotations and minimap alignment of the aerobactin-positive plasmids were then used to compare the plasmids visually with gggenomes (https://github.com/thackl/gggenomes/) version 0.9.5.9000 in R.

### Detection and characterization of the composite transposon

The 16 aerobactin-harbouring plasmids, in addition to the three reference plasmids mentioned above, were compared to two plasmids from previous studies that investigated *K. pneumoniae* harbouring aerobactin in pigs [12, 15]. However, only short reads were available from these studies. Therefore, these were compared to the rest of the above sequences on a gene level. Reads from one sample from each study (accession numbers SAMN07319199 and ERR3932286 for Germany and Italy, respectively) were downloaded and quality-checked before being assembled as described above. The draft genomes were subjected to VirulenceFinder, using the extended database, to identify the contig harbouring aerobactin. This contig was subsequently annotated using Bakta. The genetic neighbourhood of the aerobactin operon was manually scanned using the gff3 file from the annotation for all the 21 sequences. Potential composite transposons and other mobile elements were detected by using MobileElementFinder [43] version 1.0.3, database version 1.0.2, and the results were compared to the manual investigation. The detected composite transposon harbouring the aerobactin operon was extracted from the plasmid fasta sequence using Seqkit, and annotated with Bakta as described above, excluding the --circular option. ISFinder [44] blast was used to characterize the potential insertion sequence (IS) elements flanking the putative composite transposon. The IS elements that were closest to the genetic coordinates of the putative composite transposons were selected. If ties occurred, the highest scoring result was selected based on the blast results.

To confirm the presence of the composite transposon in the aerobactin-harbouring samples that were not long-read sequenced, the raw reads were mapped to a representative sequence of the composite transposon. This was performed in the Ellipsis pipeline by mapping with bwa [45] version 0.7.17 and SAMtools [46] version 1.9.

To determine the phylogenetic relationship between the composite transposons, ParSNP [47] version 1.6.1 was used to generate an alignment, using one of the input sequences as a reference at random, followed by a phylogenetic inference with iq-tree with the same settings as described above. Snp-dists was used to generate SNP distances from the ParSNP alignment.

### blast search

The composite transposon sequences were subjected to a blastn search to identify the presence of the sequence in other bacterial species, or from *K. pneumoniae* from other hosts, by using blast+ [48] version 2.9.0. blast+ was used to run a search for the c-type lysozyme inhibitors *ivy*, *pliC* and *mliC* (accession numbers WP_004178946, ATI89757 and WP_032448305, respectively). The *mliC* gene was selected due to already being present in the composite transposon. The remaining two genes were selected since they had previously been identified in several *Klebsiella* species [49]. The fasta file with the three gene sequences was used as the subject, and each of the 233 draft assemblies as the query, using default settings.

Any hypothetical proteins or gene sequences of interest in the composite transposon were subjected to a blastx search on the blast website using default parameters. A representative gene sequence of each gene was selected based on a quick codon-aware alignment in mega-x [50] version 10.0.5 using muscle.

## Results

### Detection and identification of *K. pneumoniae*

Of the 299 investigated pig caecal samples, 155 (52.0 %, 95 % confidence interval 46.0–57.6) were culture positive for *Klebsiella* spp*.* From each positive sample, one isolate of *Klebsiella* spp. was included for further investigation. MALDI-TOF MS identified 128 *K. pneumoniae* (82.6 %), 18 *Klebsiella variicola* (11.6 %), 7 *Klebsiella oxytoca* (4.5 %) and 2 *Klebsiella aerogenes* (1.3 %) isolates among these. In total, isolates from 146 samples (48.8 %, 95 % confidence interval 43.0–54.6) were classified as part of the KpSC. All non-KpSC isolates were excluded from further analysis.

Among the 146 KpSC isolates, 128 (87.7 %) were *K. pneumoniae sensu stricto*, 16 (11.0 %) *K. variicola* subsp. *variicola* and 2 (1.4 %) *Klebsiella quasipneumoniae* subsp. *similipneumoniae* using whole-genome sequencing and Kleborate v. 2.1.0. See Table S1 for the full results from Kleborate.

### Virulence and resistance gene identification

The genomes of the 146 KpSC pig isolates from Norway were compared to 87 KpSC genomes from pigs in Thailand, which were re-run on Kleborate. Altogether, virulence determinants were detected in 114 out of the 233 isolates (49.0 %).

**Fig. 1.**
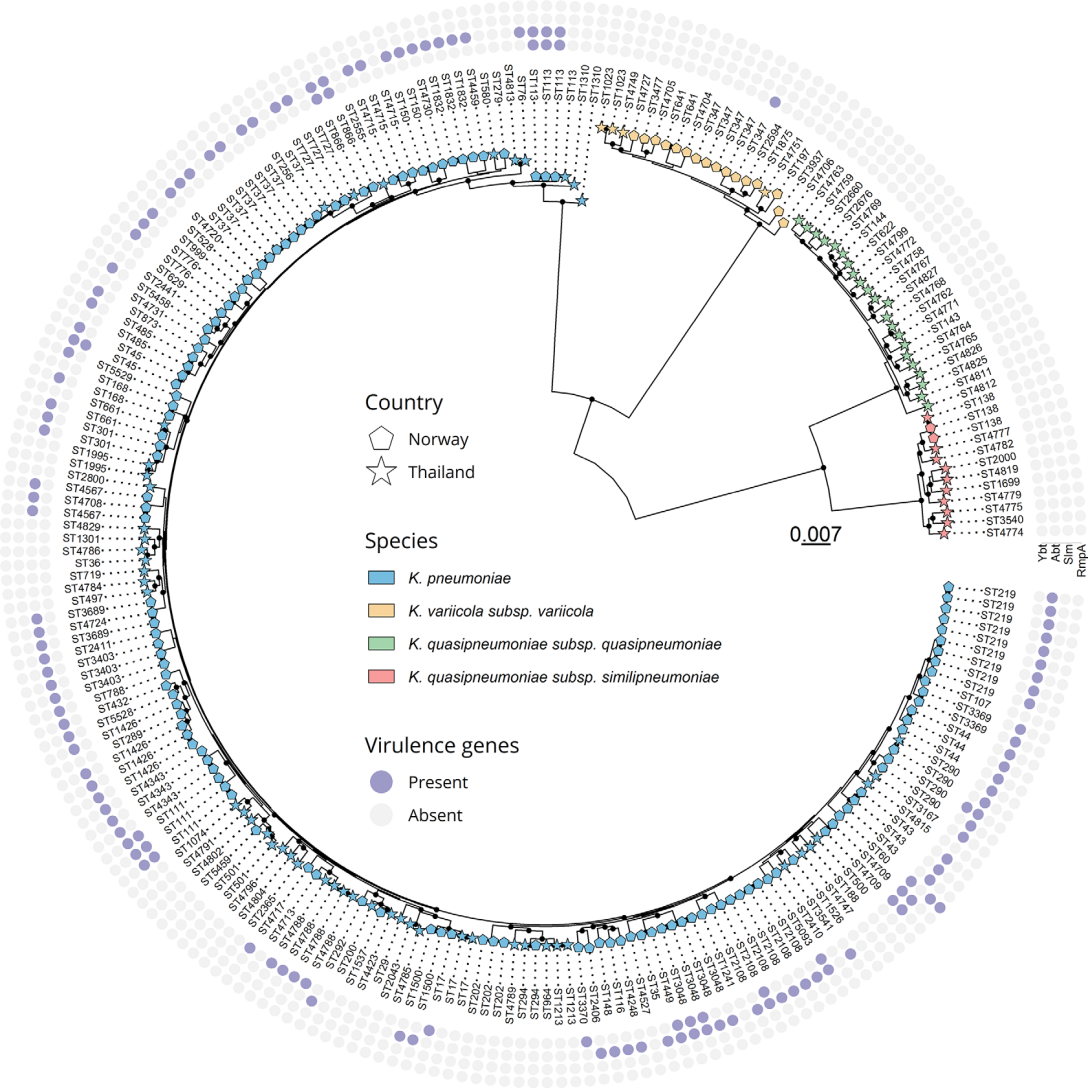
Core-gene phylogeny and virulence determinants of *Klebsiella* spp. isolates. Phylogenetic tree based on an alignment of 3875 core genes from the 233 *Klebsiella* pig isolates from Norway (pentagons) and Thailand (stars). Black dots on nodes represent accepted bootstrap values (≥95). Subspecies of *K. pneumoniae* are represented by colours on the tips. Each ST is presented as a tip label. Presence/absence of specific virulence genes/operons is presented on the four outermost rings: Ybt, yersiniabactin; Abt, aerobactin; Slm, salmochelin; RmpA, *rmpA/rmpA2*. Model of evolution: GTR+F+R10. Tree scale shows substitutions per site.

Virulence determinants were detected in 106 (72.6 %) of the 146 isolates from Norway, of which 101 (69.2 %) harboured aerobactin *iuc3*. Table 1 summarizes the subspecies and the occurrence of virulence determinants detected in this study. Aerobactin was exclusively detected in *K. pneumoniae sensu stricto* isolates, for both countries. The most prominent AbSTs among the isolates from Norway were AbST88 (*n*=20), AbST82 (*n*=15), AbST25 (*n*=13) and AbST83 (*n*=10). Yersiniabactin was detected in 22 isolates (15.0%). Ten different lineages of yersiniabactin were detected, the most prominent being *ybt10* ICE*Kp*4 (*n*=5), *ybt18* ICE*Kp*15 (*n*=3), *ybt19* ICE*Kp*16 (*n*=3) and *ybt8* ICE*Kp*3 (*n*=3). Salmochelin and *rmp3* was only detected in a single isolate (0.7 %). Co-localization of aerobactin and yersiniabactin was detected in 17 (11.6 %) of the isolates from Norway (Fig. 1).

**Table 1.** Overview of assigned species and per cent occurrence of virulence determinants in KpSC isolates from pigs in Norway and Thailand The species assignment for the isolates from Thailand was extracted from a previous study by Leangapichart *et al.* [16].

Species		Virulence gene (%)			
	*N*	Abt	Ybt	Slm	RmpA
**Norway**					
K. pneumoniae*	128	78.9	16.4	0.8	0.8
*K. similipneumoniae*†	2	0.0	0.0	0.0	0.0
* K. variicola* ‡	16	0.0	6.3	0.0	0.0
**Thailand**					
* K. pneumoniae *	51	7.8	7.8	0.0	0.0
* K. quasipneumoniae* §	22	0.0	0.0	0.0	0.0
*K. similipneumoniae*†	10	0.0	0.0	0.0	0.0
* K. variicola *	4	0.0	0.0	0.0	0.0
**Total**	233	45.1	11.2	0.4	0.4

Abt, aerobactin; Slm, salmochelin; Ybt, yersiniabactin.

*K. pneumoniae sensu stricto.

†*K. quasipneumoniae* subsp. similipneumoniae*.*

‡*K. variicola* subsp. variicola*.*

§*K. quasipneumoniae* subsp. *quasipneumoniae*.

Overall, 9 (6.2 %) of the 146 Norwegian isolates carried resistance genes, mainly *strA* (*n*=4), *strB* (*n*=3) and *bla*_SHV-178_ (*n*=3). Six (66.6 %) of these isolates carried aerobactin *iuc3*, in addition to *strA/B* (*n*=1), *strA/B+tetB* (*n*=1), *bla*_SHV-178_ (*n*=3), and *bla*_SHV-52_ (*n*=1).

Among the isolates from Thailand, virulence determinants were detected in eight isolates (9.2 %), where aerobactin *iuc3* was detected in four isolates (4.6 %). The detected AbSTs were AbST88 (*n*=2), AbST25 (*n*=1) and AbST13-2LV (*n*=1), some of which were also detected among the Norwegian isolates. The other four isolates harboured yersiniabactin *ybt4*, likely mobilized on a plasmid.

### Multilocus sequence typing

A total of 152 unique STs were detected among the 233 isolates [Simpson diversity index (SDI) 0.994], and 112 (74.0 %) of these STs were represented only by a single isolate. The number of unique STs among isolates from Norway and Thailand was 82 and 78, respectively (SDI 0.987 and 0.997, respectively). The most prominent STs were ST219 (*n*=9, all *iuc3* positive), ST37 (*n*=8, 5 *iuc3* positive, 3 negative), ST2108 (*n*=6, all *iuc3* positive) and ST347 (*n*=5, all *iuc3* negative), all of which were Norwegian isolates except one ST37 isolate from Thailand. Seven different STs were represented with isolates from both countries, namely ST113, ST727, ST37, ST661, ST17, ST294 and ST290.

### Pangenome analysis and phylogeny

A total of 21 343 genes were detected among the 233 included isolates. Among these, 3875 were classified as core. A core-gene alignment was generated, with a size of 3.9 Mbp. After removing constant sites, 648 kbp remained (16.5 %). A phylogenetic tree was reconstructed using the variable sites alignment (Fig. 1). ModelFinder plus detected GTR+F+R10 as the evolutionary model with the best fit. The tree consisted of four distinct clades, one for each subspecies. The *K. pneumoniae sensu stricto* clade had several deep-branching subclades with a median SNP distance of 24 374±3193 and a range of 0–37 319. The phylogeny revealed that aerobactin was not fixed to one ST or tightly clustered clade, but rather was identified throughout the *K. pneumoniae sensu stricto* clade.

### Plasmid detection and typing

**Fig. 2.**
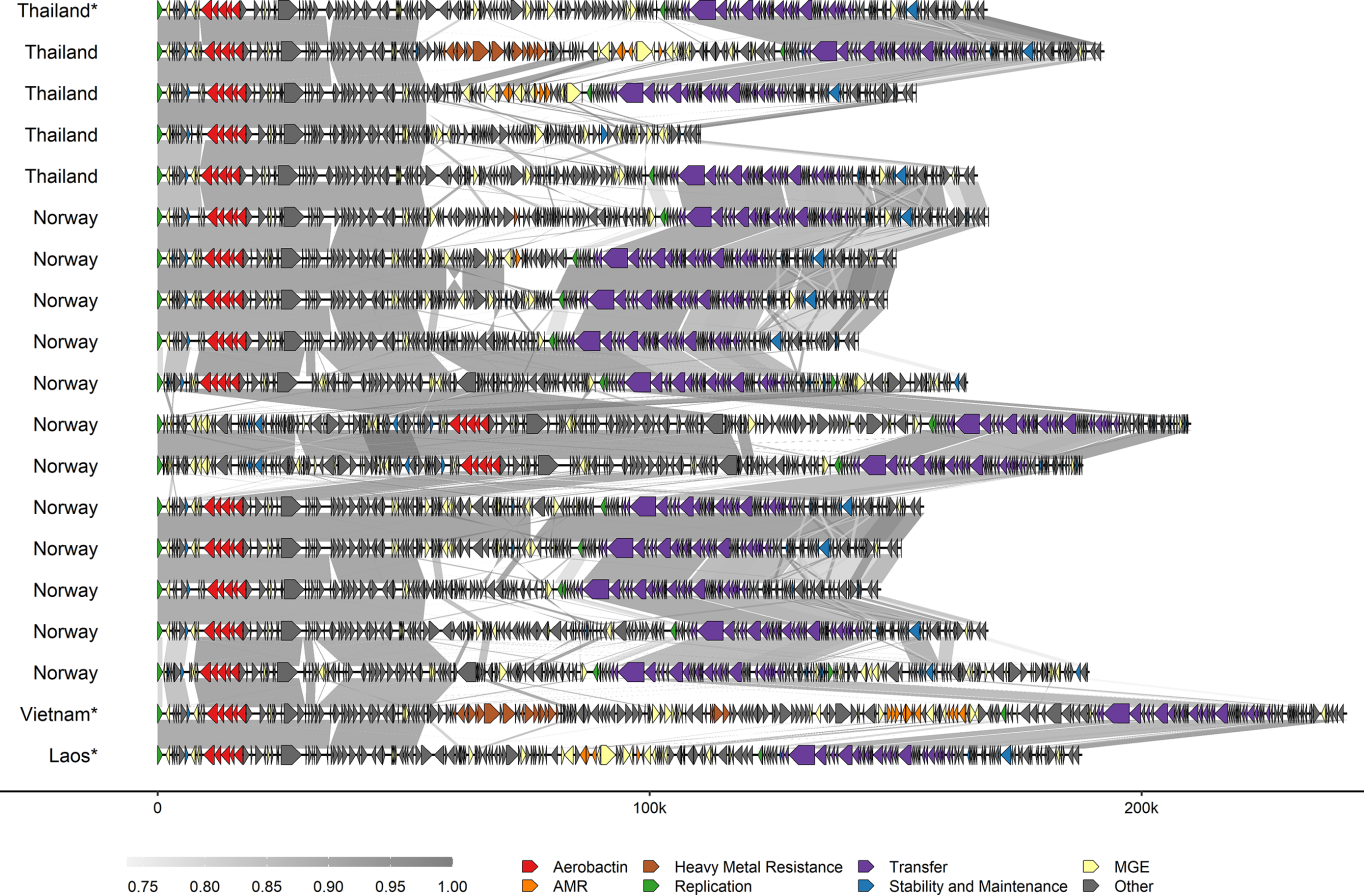
Comparison of the IncFIB_K_/FII_K_ plasmids harbouring aerobactin from *K. pneumoniae* from healthy pigs in Norway and Thailand. Reference plasmids from Thailand, Laos and Vietnam are marked with stars. Grey-shaded areas between the plasmids represent the per cent identity. Genes are marked as arrows, and the colours represent the gene functions. MGE = Mobile genetic element.

A subset of 16 isolates were selected for Nanopore sequencing to capture the diversity in the aerobactin-positive isolates. The Nanopore sequencing enabled in-depth plasmid characterization. MOB-suite detected 17 different complete plasmids and 1 incomplete plasmid among all 16 complete genomes. Aerobactin was detected on IncFIB_K_/IncFII_K_ plasmids in 15 isolates, and on a multi-replicon IncFIB_K_/R plasmid in 1 isolate from Thailand (Table S2). IncF RST showed that the IncF plasmids were of different replicon STs, the major being IncFII_K_[K9:A^−^:B^−^] (*n*=6, Table S2). The aerobactin-harbouring plasmids had a mean size of 163 086 bp. Highly conserved areas flanked the aerobactin operon in all plasmids (Fig. 2).

### Composite transposon characterization

**Fig. 3.**
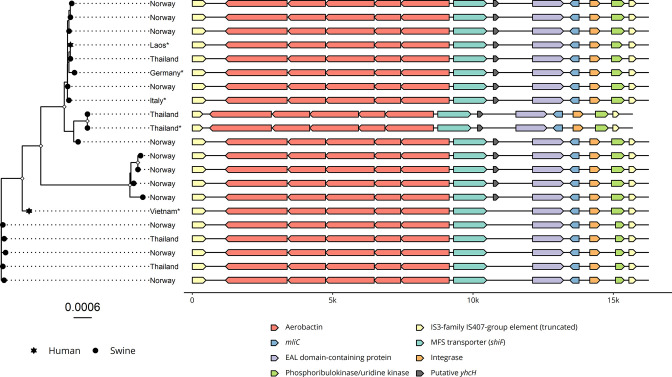
Overview of the aerobactin-harbouring composite transposon identified on IncFIB_K_/FII_K_ plasmids in *K. pneumoniae* from pigs in Norway and Thailand. To the left is a maximum-likelihood phylogenetic tree showing the evolutionary relationship between the composite transposons. The shapes on the tips represent the host species, and the asterisks the reference sequences. White diamonds on the nodes in the tree represent accepted bootstrap values (≥95). Tree scale shows substitutions per site. To the right is a schematic representation of the genetic content of each composite transposon, where colours represent the different genes.

A highly conserved putative composite transposon with a mean size of 16 213 bp harbouring the aerobactin operon was detected on all plasmids, including the reference plasmids from Thailand, Laos, Vietnam, Germany and Italy (Fig. 3, Table S3). The composite transposon was flanked by truncated IS ISKpn78-like elements, belonging to the IS*3*-family and IS*407*-group with a complete length of 1221 bp. On average, the ISKpn78-like element was truncated to 39.6 and 22.3 % of its complete length, on the left and right side, respectively.

Several genes, such as a Major Facilitator Superfamily (MFS) transporter, a c-type lysozyme inhibitor *mliC* and a hypothetical protein were detected in 15 of the transposons. A blast search with the MFS-transporter sequence revealed that it had a 90.7 % sequence identity to *shiF* [National Center for Biotechnology Information (NCBI) accession number EPF45641.1]. To identify whether the c-type lysozyme inhibitor was unique to the aerobactin-positive samples, and to identify whether the isolates harboured other lysozyme inhibitor genes, a blast search was conducted using *mliC*, *pliC* and *ivy* as subjects and the 233 draft genomes as queries. These genes encode lysozyme inhibitors that have previously been identified in *Klebsiella *, and the blast results revealed that the *mliC* gene was indeed unique to the aerobactin-positive isolates, while *ivy* was present in 232 of 233 isolates (99.6 %), and *pliC* was not present in the dataset. The hypothetical protein of the composite transposon was blast searched against the nucleotide database, and had a 100 % identity and 98 % query coverage to ‘putative protein YhcH’ (NCBI accession number QTK28692.1). This gene was present in 15 (71.4 %) of the 21 investigated composite transposons.

Read mapping of the 105 aerobactin-positive isolates to a representative sequence of the 16 kbp composite transposon revealed a median coverage of 100 % (sd 0.4), and a median reads per base value of 80 (sd 33). A blastn search of the entire composite transposon sequence revealed several hits with an identity >99 % and length >15 kbp for plasmids from *K. pneumoniae *. These *K. pneumoniae* isolates originated mostly from humans and swine in China, but also from other countries such as the UK, the USA, Thailand and Laos (Table S4). Two hits were from *K. pneumoniae* isolates isolated from humans with bloodstream infections in Norway.

Four monophyletic groups with ultrafast bootstrap support ≥95 were identified with phylogenetic analysis of the composite transposons (Fig. 3), with a mean sequence coverage of 96.6 %. Pairwise distance variation was low within each monophyletic group, with a median SNP distance of 4 and a range of 0–23. iq-tree detected HKY+F+I as the closest evolutionary model.

## Discussion

In this study, we identified aerobactin *iuc3* in a major proportion of *K. pneumoniae* isolated from the intestinal flora of healthy pigs in Norway, all harbouring a highly conserved putative composite transposon. Aerobactin *iuc3* seem to be connected to *K. pneumoniae* from pigs in other countries as well [12, 15], and comparison to data from other countries in Europe and South-East Asia showed that the composite transposon was present on diverse IncFII_K_/FII plasmids. The presence of aerobactin throughout the *K. pneumoniae sensu stricto* clade and across several STs indicates movement of the plasmid between *K. pneumoniae* from different genetic backgrounds. The findings in this study may help elucidate the potential reservoir of this virulence determinant.

A high occurrence of *K. pneumoniae* carriage in healthy pigs in Norway was detected in the current study, where almost 50 % of the samples were positive. A limited number of studies have investigated the occurrence of *K. pneumoniae* and *Klebsiella* spp. in pigs, but these few studies have identified a similar carriage rate [15, 16]. We have previously detected *K. pneumoniae* in 25.8 % of broiler and 74.2 % of turkey flocks in Norway [51]. Comparisons to the occurrence found in the present study, however, cannot be made directly as the present study on pigs analysed one individual animal sample per pig herd, while the previous broiler and turkey study analysed pooled samples of ten animals per flock. In healthy humans in Norway, a study investigating the gastrointestinal carriage of *K. pneumoniae* identified an occurrence of 16.3 % [52]. Although differences in study design, sampling and methodology must be taken into account, the high occurrence in animals, especially in pigs and turkeys, compared to humans indicates a host-specific carriage of *Klebsiella* in the gut. Moreover, a high ST diversity was detected among the *K. pneumoniae* from pigs. This ST diversity is reflected in the phylogenetic tree, where several deep-branching lineages are prominent within each of the subclades. Similar findings have previously been described among *K. pneumoniae* in both humans and animals in several countries [12, 13, 16, 52, 53]. This level of diversity indicates that the *K. pneumoniae* population in the gut of Norwegian pigs is composed of a variety of strains, and that no specific lineage is dominant.

A major proportion of the *K. pneumoniae* harboured aerobactin *iuc3*. This is in concordance with the studies on *K. pneumoniae* from pigs in Germany and Italy [12, 15]. In contrast, aerobactin seems to be less prominent in *K. pneumoniae* from healthy poultry and humans in Norway. Among the poultry isolates, 7.4 % carried aerobactin *iuc5* [51]. Moreover, aerobactin was detected in only 7 (2 of which were *iuc3*) out of 484 intestinal *K. pneumoniae* isolates from healthy humans in Norway (1.4 %) [52]. Another Norwegian study has described the finding of *iuc3* in *K. pneumoniae* from two humans with bloodstream infections [54]. A blast search identified the composite transposon sequences in these two isolates, as well as in *K. pneumoniae* isolates from China, Thailand, the UK, the USA and Laos, mostly from pigs and humans. Taken together, this suggests that pig KpSC populations may be a reservoir for these composite transposons, or more precisely IncFIB_K_/FII_K_ plasmids harbouring the composite transposon. The identification of the putative composite transposon in *K. pneumoniae* from human clinical samples suggests that it might play a role in infections with *K. pneumoniae.* However, a recent study suggests that there is little evidence for zoonotic transfer of *K. pneumoniae* between the pig and human reservoirs, and that transmission between niches is less frequent than within niches [15]. The study also states that the transmission dynamics of plasmids is likely different than that of whole-bacterium transmission. Thus, the observation of the putative composite transposon within a human clinical isolate may, therefore, be attributed to transfer of the plasmid itself rather than strain transmission.

Interestingly, a major difference in the occurrence of aerobactin was detected among pig isolates from Norway and Thailand. This difference, however, may be due to differences in both animal husbandry practices and study design. The isolates from Norway originated from fattening pigs, while the isolates from Thailand mainly originated from sows. Also, the Norwegian samples were caecal samples, one sample per herd, while the Thai samples were rectal swab samples, with up to ten sampled animals per herd. The Norwegian study was designed to be representative for the Norwegian pig population, while the study from Thailand was restricted to 164 farms in the Khon Kaen province in Northern Thailand. Another explanation for this difference in occurrence of *iuc3* may be the higher proportion of non-* K. pneumoniae* subsp. *pneumoniae* isolates in the dataset from Thailand, as *iuc3* seems to be highly associated with this subspecies in the current study.

Aerobactin *iuc3* has previously been associated with the dissemination of diverse IncFIB_K_/FII_K_ plasmids [11, 55]. In the current study, *iuc3* was identified on IncFIB_K_/FII_K_ plasmids with different IncF RST profiles and in several different *K. pneumoniae* STs. IncFIB_K_/FII_K_ plasmids harbouring aerobactin have previously been confirmed as conjugative [56, 57], and the acquisition of the plasmid has been shown to enhance the virulence in both *K. pneumoniae* and *Escherichia coli* [56]. The mobility potential of these plasmids is a cause for concern, as virulence may spread within or across species. Since aerobactin-positive isolates were identified throughout the *K. pneumoniae sensu stricto* subclade in the phylogenetic tree, the plasmids have likely moved between isolates with different genetic backgrounds within the Norwegian pig population. The aerobactin operon was identified on a highly conserved putative composite transposon. The presence of truncated IS elements flanking the putative composite transposon indicates that the transposon has been permanently integrated into the plasmid, and is likely no longer mobile. However, further investigation is needed to confirm this. A possible mobilization will represent an additional risk of virulence transmission and convergence events, as it may allow for the aerobactin operon to mobilize and integrate into a plasmid already harbouring resistance genes. Convergence events have possibly occurred in the plasmids from Thailand, as these also harboured antibiotic-resistance genes that confer resistance towards, among others, tetracycline, trimethoprim, aminoglycosides, (fluoro)quinolones and sulfonamides [16].

The aerobactin operon was co-localized with several additional genes in the putative composite transposon. For example, the MFS transporter that is located next to the aerobactin operon had a high sequence identity to *shiF*, which is hypothesized to be physically and functionally linked to aerobactin in *E. coli* and to increase aerobactin secretion [58]. In addition, the putative composite transposon uniquely harboured *mliC,* which encodes a c-type lysozyme inhibitor [59] that is a lysozyme produced by several mammals, including pigs [60]. In addition to the seemingly ubiquitous *ivy* lysozyme inhibitor, the presence of *mliC* in the aerobactin-positive isolates may provide further protection against c-type lysozyme action, which may give them a fitness advantage in mammalian hosts. Another co-localized gene encoded an EAL-domain containing protein, which may be involved in the hydrolysis of c-di-GMP, regulating processes such as virulence and fimbral expression and biofilm formation [61, 62]. Lastly, a gene encoding a putative YhcH protein was present in the majority of the investigated putative composite transposons. This protein has previously been linked to interactions with sialic acid in *Haemophilus influenzae* and *Helicobacter pylori* [63, 64], and is a compound that can be used as a nutrient source, and which is linked to immune system evasion [65]. The presence of these genes on the putative composite transposon may provide additional virulence potential to the host bacterium other than iron scavenging by the siderophore aerobactin. However, further studies are needed to confirm the effects of these genes with regards to virulence and fitness.

In conclusion, our data indicate that *iuc3* is part of a putative composite transposon located on IncFIB_K_/FII_K_ plasmids. The putative composite transposon harbours additional genes that may enhance virulence and/or fitness in mammalian hosts. The high occurrence of aerobactin-positive *K. pneumoniae* in Norwegian pigs, taken together with international data, suggests that pigs are a reservoir for *K. pneumoniae* with *iuc3.* The presence of truncated IS elements flanking the composite transposon indicates that the transposon has become permanently integrated into the plasmid and is likely no longer mobile. Further investigations into the sources and mobility of the putative composite transposon, and the association with pig KpSC, is warranted to confirm that pigs might be a reservoir for *K. pneumoniae* harbouring aerobactin *iuc3*.

## Supplementary Data

Supplementary material 1

Supplementary material 2

Supplementary material 3

Supplementary material 4
